# Influence of insolvency of a football club in Poland on the right to participate in league games

**DOI:** 10.1007/s40318-020-00178-4

**Published:** 2020-12-15

**Authors:** Rafał Adamus

**Affiliations:** Katedra Prawa Gospodarczego i Finansowego Uniwersytet Opolski, Opole, Poland

**Keywords:** Football club, License, Bankruptcy, Restructuring, *Lex sportiva*

## Abstract

The study is devoted to the issue of the impact of sports club insolvency in Poland on the right to participate in league games. In this respect, not only the regulation of insolvency law is of the key importance. There are also crucial organizational rules established by the sports association (the Polish Football Association—Polski Związek Piłki Nożnej) determining admission to league games of only such sports clubs which have adequate financial liquidity. It should be stressed that the Polish Football Association (hereinafter “PZPN”) regulations do not allow to take part in competitions sports club in bankruptcy. This topic has become particularly relevant in 2020 in the context of the economic consequences of the unexpected pandemic of SARS-CoV-2. The paper deals with some legal aspects of *lex sportiva* and with the basic model of organization of the professional football league in Poland as well.

## Introductory issues

At the beginning of the analysis, it should be presented the most important information on the method of organization of sport activities in Poland.

Sport^1^ activities in Poland, including football, are conducted in particular in the form of a sports club (Article 3 sec. 1 Act on sport^2^). The sports club operates in legal transactions as a legal entity (Article 3 sec. 2 Act on sport). It should be explained that—in accordance with Article 15 sec. 3 Act of sport—sports clubs operating in the professional league should be either a joint-stock company or a limited liability company. However, point 4.1.2.1. of the License Guide^3^ states that football clubs participating in the “Ekstraklasa” games (the highest football class in Poland) must have the legal form of a joint-stock company^4^ which is the most formalized company form in Polish commercial law.

It should also be added that in a sport in which competition is organized in the form of a league, the Polish sports association may create a professional league. And if more than half of the sports clubs participating in the league competitions in the highest competition class in a given sport operate in the form of joint-stock companies, it is a legal obligation to create a professional league (Article 15 sec. 1–2 Act on sport)^5^. PZPN is a founder of the professional football league in Poland.

The professional league is managed by a legal person operating as a commercial company. The rules of its functioning are set out in the agreement concluded between the Polish sports association and the league management company. The agreement should contain, in particular, provisions guaranteeing the implementation of the rights of the Polish sports association, including in the scope of establishing and implementing sports, organizational and disciplinary rules and its share in revenues related to the management of the professional league (Article 15 sec. 4–5 Act on sport).

Sports clubs in Poland, operating in the form of commercial companies (a limited liability company or a joint-stock company or theoretically in other forms^6^), have - under the Restructuring Law Act^7^ and Bankruptcy Law Act^8^—restructuring and bankruptcy capacity. The same should apply to clubs operating in the form of associations with legal personality and conducting economic activity, but they do not exist in “Ekstraklasa”—the highest football league in Poland.

## League law—organizational rules set by sport associations

The next issue that requires a short presentation is related to the competence of the sports association to create internal norms of conduct.

The term "*lex sportiva*” /“sports law” is commonly used in the theory of law^9^. It is assigned various ranges of meaning. In one of the broader meaning "*lex sportiva*” covers all the regulations and case law from non-governmental sports organizations and institutions operating in international (or supranational) space, however, the emphasis is on the special importance of separate sports principles (*principia sportiva*)^10^.

Regulation of sport matters by sports organizations enjoys considerable autonomy in Poland^11^. In accordance with Article 13 sec. 1 point 2 Act on sport the Polish sports association has the sole (exclusive) right to establish^12^ and implement sporting, organizational and disciplinary rules in the sporting competition it organizes^13^. There is the only exception of disciplinary rules on doping in sport. It should be stressed that under Article 7 sec. 1 Act on sport a sports association is created to organize and manage competition in a given sport. This is the normative basis for creating the so-called league law (a type of soft law), which is not a set of generally applicable standards. On this normative basis is issued, among others PZPN License Guide^14^.

It should be expressed an opinion that sports, organizational and disciplinary rules established—in a specific legal form—by the Polish sports association are not synonymous with the standard contract template established by one of the parties of the contract, in particular the general terms of the contract, contract templates, regulations within the meaning of Article 384 § 1 of the Civil Code (hereinafter “C.C.”). They are not created on the basis of a contractual standard of general competence (Article 353^1^ C.C.), but on the grounds of the Act on sport. Sports, organizational and disciplinary rules set by the Polish sports association are—within their jurisdiction—effective *erga omnes*. The minister competent for physical culture exercises supervision over the activities of Polish sports associations (Article 16 sec. 1 Act on sport)^15^. Organizational rules set by the Polish sports association are subject to review for compliance with legal regulations (Article 16 sec. 3 Act on sport)^16^. *Lex sportiva* may not be contradictory to mandatory (*iuris cogentis*) provisions of state law.

## PZPN license system based on UEFA regulations

The PZPN License System, including the granting of licenses^17^ authorizing the licensee to participate in PZPN club competitions and UEFA club competitions, has, inter alia, the following goals: "improving the economic and financial capabilities of clubs, increasing their transparency, credibility" and "protecting club creditors by ensuring that the club fulfills its obligations to players, coaches, employees, tax / social security institutions and other clubs WZPN^18^, PZPN and UEFA" (Point 1.1. d, e of the License Guide).The license is not transferable in any way to another person (point 4.3.1.9. point c, d of the License Guide).

The License Guide focuses on protecting the interests of selected categories of creditors of the sports club. The universal *pari passu* rule is not implemented. It should be indicated here that there is prohibited—by state law in Poland - favoring creditors in the event of threat of debtor insolvency or bankruptcy. The indicated axiological foundations of the licensing system result in specific practical consequences. The license shall expire automatically without prior notice upon the issue of a court ruling on bankruptcy against the license (in accordance with point 4.3.1.3. point c, d of the License Guide).

It should be explained here that in the event of indications of insolvency, the management board of the sports club in Poland has a legal obligation to file for bankruptcy. The application is submitted to the competent commercial court. Applications with formal deficiencies that have not been corrected shall be returned by the court. Filing a bankruptcy petition initiates proceedings in respect of announcement of bankruptcy proceedings. Filing a bankruptcy petition is not a reason for expiry of the license. At this stage the court examines whether the conditions for bankruptcy have come true. Then, upon the declaration of bankruptcy, the proper insolvency proceedings begin.

In case of declaring bankruptcy of a sport club by the court the license expires automatically by the virtue of law. There is no need for any action by the licensing authority for this purpose. For the license to expire, the court's ruling need not be final. Does the license reappears if the bankruptcy order is revoked? The license rules do not specify this problem. However an expired license is not subject to restitution.

The PZPN License System is based on UEFA Club Licensing and Financial Fair Play Regulations (hereinafter “UEFA Reg.” or “UEFA Regulations”). The UEFA Regulations aim to achieve maximum level of “financial fair play” in UEFA football club competitions. They are created in order to improve the economic and financial capability of the football clubs, increasing their financial transparency and credibility (Article 2 sec. 2a UEFA Reg.). UEFA stressed the necessary importance on the protection of some kinds of creditors of the football clubs. Football clubs should settle their liabilities with employees (players), social and tax authorities and other clubs in due time (Article 2 sec. 2b UEFA Reg.). UEFA intends to provide “more discipline and rationality” in club finances (Article 2 sec. 2c UEFA Reg.). UEFA aims “to encourage clubs to operate on the basis of their own revenues” (Article 2 sec. 2d UEFA Reg.). The club's financial standing is of key importance in terms of allowing the club to participate in the competition at the national and European level. UEFA Regulations provides for the forced elimination from the football competition of clubs having serious financial problems.

UEFA Regulations uses the term “protection from creditors” with the following meaning: “procedures pursuant to laws or regulations whose objectives are to protect an entity from creditors, rescue insolvent entities and allow them to carry on running their business as a going concern. This process encompasses administration procedures and other insolvency proceedings (that might result in a compromise with creditors, bankruptcy or liquidation).” Those proceedings are stipulated by state law (*lex concursus*).

In Annex IX UEFA Reg. “Licensor’s assessment procedures”, it is clearly stipulated that if the license applicant or any parent company of the license applicant included in the reporting perimeter is or was seeking protection or has received or is still receiving protection from its creditors pursuant to laws or regulations within the 12 months preceding the license season then the license must be refused. For the avoidance of doubt the license must also be refused even if the concerned entity is no longer receiving protection from its creditors at the moment the licensing decision is taken.

## Economic risks of sports clubs: insolvency risk

Professional sport is a high-risk commercial activity. Financial outlays do not always translate into a sports result, and the latter has a proportional impact on the volume of funds raised due to audiovisual rights, payments from sponsors, revenues from the match day, etc. Sometimes an accidental player's injury scheduled for the transfer may cost the selling club huge sums of money. Problems with the proper liquidity of the sports club or its excessive debt are unfortunately an integral part of the professional sport landscape^19^.

In professional sport players' salaries, fees for transferring players from another club have rapidly grown up till COVID 19 crisis. The global trend does not bypass Polish sports clubs. However, Polish clubs cannot match the financial potential of the richest European football clubs^20^. Polish sports clubs have private owners (Lech Poznań, Lechia Gdańsk, Legia Warszawa etc.). However, many clubs are municipal companies maintained by municipalities (Piast Gliwice, Górnik Zabrze, Śląsk Wrocław, etc). The amounts of public subsidies are rather small for financial needs^21^. Polish clubs most often did not bear the costs of stadium construction. Stadiums are usually the property of municipalities. Clubs use stadiums based on rental agreements.

As a rule, the biggest expense for football clubs is player salaries. Neither FIFA nor UEFA has introduced solutions limiting expenditure on wages for players („salary cap” or „wage cap”)^22^. Salary cap can be expressed as a limit for a single player or for a whole team or a limit for both. In practice, this means that sports clubs have to build very large budgets. Too high salaries seems to be the most common cause of insolvency^23^. In Poland, players are usually not employed under an employment contract. The basis of relations with the club is civil law contracts. The principle of being subject to social security raises a lot of ambiguities in practice.

In recent years, there have been in Poland several spectacular—in the local dimension—bankruptcies of football sports clubs. It should be mentioned^24^ Odra Wodzisław (2011r.), Widzew Łódź (2015), Polonia Warszawa (2018). Some clubs were in restructuring proceedings: Ruch Chorzów (2017). Some clubs were in such a bad financial condition that they filed for bankruptcy: ŁKS Łódź (2012) Śląsk Wrocław (2013). This issue has been generally described in the Polish literature^25^.

The SARS-CoV-2 pandemic caused very serious problems for financial stability of sports clubs^26^. Canceling the games means reducing revenues from audiovisual rights, lower revenues from sponsors and advertisers, cutting-off revenues from ticket sales, etc. Many sports clubs have faced the risk of insolvency since Spring 2020^27^.

## Expiry of the license in case of bankruptcy

The content of league law regarding the insolvency of a sports club prompts some reflection. The following question arises: can the Polish sports association validly stipulate in its league law that the declaration of bankruptcy of the sports club results in the loss of the right to participate in the games?

*Prima facie* the issue of the admissibility of such provisions is important for practice. A football club whose license has expired may not continue participating in the competition. Loss of license has a significant impact on the legal existence of contracts with players and the training staff.

First of all contracts with players are subject to league law. To an extent not regulated in league law, the contract should be governed by the applicable state law. The specificity of sport is of great importance as well^28^. In accordance with CAS jurisprudence^29^, the interpretation of the FIFA rugulations should be made in accordance with Swiss law. In the opinion of CAS this solution should ensure an uniform interpretation of the FIFA rules. Aspects which are not regulated in the applicable FIFA rules may be subject, subsidiarily, to foreign applicable law. Under swiss law, players are generally considered as employees. It is stipulated in league law that a contract between a professional player and a club may only be terminated upon expiry of the term of the contract or by mutual agreement (Article 13 Regulations on the Status and Transfer of Players, hereinafter „RSTP”). A contract may be terminated by either party without consequences of any kind (either payment of compensation or imposition of sporting sanctions) where there is „just cause” (Article 14 sec. 1 RSTP). An established professional who has, in the course of the season, appeared in fewer than ten per cent of the official matches in which his club has been involved may terminate his contract prematurely on the ground of sporting just cause. Due consideration shall be given to the player’s circumstances in the appraisal of such cases. The existence of sporting just cause shall be established on a case-by-case basis. In such a case, sporting sanctions shall not be imposed, though compensation may be payable. A professional may only terminate his contract on this basis in the 15 days following the last official match of the season of the club with which he is registered. (Article 15 RSTP).

However, Article 7 section 1 Regulation (EU) 2015/848 of the European Parliament and of the Council of 20 May 2015 on insolvency proceedings (hereinafter „Reg. EU”) stipulates that save as otherwise provided in this Regulation, the law applicable to insolvency proceedings and their effects shall be that of the Member State within the territory of which such proceedings are opened (the ‘State of the opening of proceedings’—*lex concursus*). The law of the State of the opening of proceedings shall determine the effects of insolvency proceedings on current contracts to which the debtor is party (Article 7 section 2e Reg.EU). If the *lex concursus* is Polish law Article 102 section 1 B.L. should be appliacable. According to this the mandate contract concluded by the bankrupt, in which the bankrupt was the principal expires on the day of declaration of bankruptcy. Claim for the resulting loss may be claimed in bankruptcy proceedings.

It should be marked that in accordance with Article 83 of Bankruptcy Law Act (hereinafter “B.L.”) provisions stipulating that in the event of submission of a bankruptcy petition or in the event of a declaration of bankruptcy by court ruling, a change or termination of the legal relationship to which the party is bankrupt is null and void. The provision of Article 83 B.L. is designed to secure the proper conduct of insolvency proceedings. The provisions relating to the contract should be applied *mutatis mutandis* to resolutions and other multilateral legal actions as well as to unilateral legal actions.

Thus, the provision of the contract reserving a change or termination of the legal relationship to which the party is a bankrupt is invalid in the event of: (a) filing for bankruptcy or (b) declaration of bankruptcy.

However, the critical event is not a "threat of insolvency", but the contractual provisions reserving a change or termination of the legal relationship in the event of a restructuring application are void due to other legal grounds (Article 247 of the Restructuring Law Act hereinafter “R.L.”) regarding accelerated arrangement proceedings, Article 247 R.L. in connection with Article 273 R.L. regarding arrangement proceedings, Article 247 in connection with Article 297 R.L. regarding sanation proceedings.^30^.

It should be defended the view the clause stipulated by *lex sportiva* according to which a sports club's bankruptcy declaration results in the loss of the right to participate in the competition is not null and void. On the contrary it remains in force.

The provision of Article 83 B.L. refers to "contract terms". It was placed among the provisions regulating the impact of bankruptcy on the civil "obligations" of the bankrupt. In principle, except in the case of Article 86 B.L., these provisions apply either to contract law (ineffectiveness, offsetting, interest withdrawal from the mutual contract, transformation of non-monetary liabilities into monetary, immediate maturity, etc.), or to specific types of liabilities (e.g., rent, lease, order, etc.) in the context of bankruptcy. On the other hand, the License Guide clause on the termination of the license in the event of the bankruptcy of a sports club is not a "contractual provision". Its specific legal nature has already been highlighted.

Moreover, this clause refers to relations related to participation in the game system. Therefore, it is of a legal and organizational nature—it regulates the specific relation of participation in a specific sporting venture. In the background of this argumentation, it should be remembered, for example, the effects of bankruptcy of a commercial partner of a partnership for the company's relationship or the effects of bankruptcy of a partner in a civil law partnership in Poland. The legislator allows for the annihilation of a legal-organizational relationship in the event of bankruptcy.

It must not be forgotten that sufficient level of solvency is needed to run a sports club without any disruption. The elimination of a joint-stock company in bankruptcy from the league is axiologically justified.

There is one more argument in the discussion. A potential buyer of an enterprise of a bankrupt sports club or an organized part of such enterprise (as part of, for example, prepared liquidation, Article 56a B.L.) can individually obtain a license which is "not transferable". This argument—in the current state of league regulations—is only theoretical due to the competition calendar and the licensing process calendar. For the future, it seems that the License Guide should introduce a fast license qualification path (including the flexible design of the license promise) for the buyer of a bankrupt sports club enterprise. Bankruptcy, as general enforcement proceedings, is the best way to protect the interests of all creditors of an insolvent debtor and in this space the regulation of league law cannot compete with generally applicable state law. The fear of being excluded from the competition may prevent the sports club board from submitting an obligatory bankruptcy petition. Thus, the inflexible regulation of the License Guide may lead to some side effects contrary to the axiology of the licensing system: the lack of timely application for bankruptcy of the sports club will work against its creditors. It seems that it is possible to reconcile the legal and economic security of the creditors of the insolvent sports company and to ensure the continuity of sports games.

## Effects of opening the restructuring procedure

The regulation of licensing rules for participation in league games should refer to the effects of opening restructuring proceedings.

In accordance with Article 2 of the Restructuring Law, the debtor is restructured in the following four restructuring proceedings: in arrangement approval proceedings, in accelerated arrangement proceedings, in arrangement proceedings and in sanation proceedings. Proceedings for approval of the arrangement allow the conclusion of the arrangement as a result of the independent collection of votes by creditors by the debtor without the participation of the court. The accelerated arrangement procedure allows the debtor to conclude an arrangement after preparation and approval of the list of claims in a simplified manner. The arrangement procedure, in turn, allows the debtor to conclude an arrangement after preparation and approval of the list of claims. Sanation proceedings allow the debtor to carry out remedial actions and to conclude an arrangement after the inventory of receivables has been drawn up and approved.

The maintenance of the license should be considered admissible subject to the conclusion by the sports club of a restructuring (non-liquidation) agreement or the submission of arrangement proposals which do not provide for any redemption for certain categories of interest (subject to the *volenti non fit iniuria* principle).

## Summary

As a result of the above short analyses it should be pointed out that due to UEFA Regulations a sports association possesses its competence to prevent from taking part in a competition an insolvent football club. PZPN adopted those rules in Poland. Formal declaration of bankruptcy by a court is not essential; however, there is a proper reason to terminate the license. A state of factual insolvency (non-payment of its obligation) of a football club is a ground for expiry of the license as well.

The licensing system—as a part of a specific league law - introduced by the Polish sports association—PZPN probably could include more flexible and amicable for investors regulations regarding the effects of bankruptcy. The same concerns the effects of the opening of sports club restructuring for given licenses. The creditor protection that underlies the axiology of the licensing system cannot, in specific solutions, depart from the aims of insolvency law.

## Footnotes

1 Sport – with the meaning of the Act on June 25, 2010 on sport - is all forms of physical activity that through *ad hoc* or organized participation affects working out or improvement physical and mental condition, development of social relations or achievement sports results at all levels. Activity-based on intellectual competition which goal is to achieve a sports result is also considered sport

2 Act on June 25, 2010 on sport, consolidated text Journal of Laws from 2019, item 1468, as amended

3 Podręcznik Licencyjny dla Klubów Ekstraklasy na sezon 2020/2021 i następne 2020, hereinafter „Licence Guide”

4 On the financing of football clubs, see for example: Kiełbasiński and Kosecki M 2015 p. XL, Osiński 2017 p. XXIV, Warchoł (2015) p. IX

5 Krześniak 2016*passim* (habilitation thesis)

6 Simple joint stock company or *Societas Europea*

7 Adamus 2019a, b, c, d, e, f Prawo restrukturyzacyjne. Komentarz *passim*

8 Adamus 2019a, b, c, d, e, f Prawo upadłościowe. Komentarz *passim*

9 Siekmann and Soek 2012 p. 12, Siekmann 2012 p. 12, James 2013 p. 10, Blackshaw 2017 p. 8, Gardiner 2001 p. 7, Greenfield and Osborn 2000 p. 5, Widłak 2015 p. 81

10 Widłak 2015 p. 84

11 Wiktorowski D 2015a, b Trybunał Arbitrażowy ds. Sportu przy PKOL jako organ jurysdykcji w sprawach sportowych p. VIII, Chołub 2015 p. IV, Kulikowska 2015 p. XXXIII

12 Greenfield and Osborn 2001 p. 14

13 Sports rules and fair play rules in sport are referred to as *lex ludica*. Foster 2006

14 Wiktorowski D 2015 Nowe przepisy licencyjne PZPN dla klubów Ekstraklasy p. IV, Bołyń 2016 p. IV

15 The supervision of the minister competent for physical culture does not cover disciplinary and regulatory decisions of the authorities of Polish sports associations related to the course of sport competition (Article 16 sec. 2 Act on Sport). Nowakowska and Osiński 2017 p. CIX, Wiktorowski D Ustawowa regulacja i problemy praktyczne występujące przy nadzorze nad polskimi związkami sportowymi 2016 p. XXXVII

16 Some controversy in league regulations has raised, for example, by a clause giving privileges to certain creditor groups (The Football Creditor Rule). It was contrary to the principle of equal treatment of creditors (pari passu rule) Windsor and Sidle (2012), p. 1–2

17 Within the meaning of the certificate issued by the PZPN confirming that the sports club meets the minimum requirements and allows the sports club to be admitted to the PZPN club competition and / or UEFA club competition.

18 WZPN – Voivodeship Football Association

19 Serby (2014) p. 12, Beech et al. (2010) p. 236

20 For comparison: the annual budget of well managed “Piast Gliwice” - Polish football champion club from the 2018/2019 season - amounted only to PLN 25 million (about EUR 5,8 million). In turn, the annual budget of the richest Polish football club (“Legia Warszawa”) in the same period was PLN 108 million (about EUR 25 million). Adamus R 9.7.2019 Piłkarze z Gliwic piszą nie tylko historię swojego klubu ale i miasta. p. 20

21 Adamus (2020) Czy polska piłka klubowa ma szanse na doinwestowanie? p. 18 Adamus (2019) Pieniądze na boisku nie grają, ale w sporcie wygrywa ten, kto je ma p. 20

22 Staudohar 1998 p. 3, Totty and Owens (2011) p. 46

23 Szymanski (2012) *passim* However Beech et al. (2010) *passim* identify five types of insolvency in English football: „clubs that have failed to cope with relegation; failed to pay monies due to the UK government; seen ‘soft debts’ become ‘hard debts’; lost the ownership of their stadium; or have been ‘repeat offenders’.”

24 It should be distinguished the company name of a joint-stock company being a club and the name of the club under which the club appears in football games which do not have to be the same.

25 Adamus 2019a, b, c, d, e, f Niewypłacalność klubu piłkarskiego p. 108 – 111, Fornalik and Szczudlik 2019 p. 47–54

26 COVID 19 Football Regulatory Issues, April 2020, *passim,* Adamus R 9.4.2020 FIFA wreszcie zabrała głos, p. 2-3

27 Adamus (2020) Zderzenie z górą lodową p. 3

28 Article 2 of the Rules Governing the Procedures of the Players’ Status Committee and the Dispute Resolution Chamber (the FIFA Procedural Rules)

29 e.g. CAS 2016/A/4846, Amazulu FC v. Jacob Pinehas Nambandi & FIFA & National Soccer League South Africa

30 Adamus 2019a, b, c, d, e, f Bankruptcy and Restructuring Law in Poland Societas et Iurisprudentia p. 24, Adamus 2018 Grounds for opening restructuring proceedings in Poland p. 162 – 166
